# Nano-formulated pomegranate extracts with dual cytotoxic and antimicrobial activity: molecular docking and mechanistic insights into leukemia cell targeting

**DOI:** 10.1186/s12906-026-05291-9

**Published:** 2026-02-26

**Authors:** Naglaa M. Hamdy, Ahmed Ismail, Hoda S. Sherkawy, Ayman S. Yassin, Mohamed M. Amin, Hanan M. El-Tantawy, Abdulrahman M. Saleh, Nashwa El-Khazragy

**Affiliations:** 1 Department of Medicinal and Aromatic Plants, Desert Research Center, Cairo, Egypt; 2 Department of Pharmacognosy, Faculty of Pharmacy, Fayoum University, Fayoum, Egypt; 3 Department of Medical Biochemistry, Faculty of Medicine, Aswan University, Aswan, Egypt; 4 Department of Medical Microbiology and Immunology, Faculty of Medicine, Al-Azhar University, Assiut, Egypt; 5 Department of Medical Microbiology and Immunology, Faculty of Medicine, Aswan University, Aswan, Egypt; 6 Department of Pharmaceutical Chemistry, Faculty of Pharmacy, Cairo University, Cairo, Egypt; 7 Department of Clinical Pathology-Hematology and AinShams Medical Research Institute (MASRI), Faculty of Medicine, Ain Shams University, Cairo, 11566 Egypt

**Keywords:** *Punicagranatum*, Leukemia, *Streptococcuspyogenes*, Antimicrobial, Antioxidant, Cytotoxic

## Abstract

**Ethnopharmacological relevance:**

Punica granatum (pomegranate) peel is traditionally used for its antimicrobial and health-promoting properties in several cultures. Rich in polyphenols, the peel has attracted interest for its potential applications in treating infections and cancer, particularly in integrative approaches for immunocompromised patients.

**Materials and methods:**

Pomegranate peel extracts were prepared using solvents of increasing polarity, with emphasis on the ethyl acetate fraction (PPE-EA). A nano-formulated version (n-PPE-EA) was developed using a standard nano-encapsulation technique. Cytotoxic activity was evaluated in THP-1 human leukemia cells using MTT assay, flow cytometry, and biochemical analyses. Antimicrobial activity was assessed against Streptococcus pyogenes via agar diffusion. Gene expression of BCL2, PI3K, and CDK8 was measured to elucidate mechanisms of action.

**Results:**

Among all tested extracts, PPE-EA showed the strongest dual activity, reducing THP-1 cell viability by over 50% at 100 µg/mL and inhibiting *S.* *pyogenes* with a 10.5 ± 1.1 mm zone. Nano-encapsulation enhanced both effects, reducing the IC₅₀ from 1.48 ± 0.03 µg/mL to 0.19 ± 0.01 µg/mL and increasing the bacterial inhibition zone to 15.6 ± 0.5 mm. n-PPE-EA induced apoptosis, cell cycle arrest, elevated catalase activity, and reduced malondialdehyde levels. It also downregulated BCL2, PI3K, and CDK8 expression.

**Conclusion:**

The nano-formulated PPE-EA demonstrated potent cytotoxic and antimicrobial activities, with enhanced efficacy attributed to improved bioavailability and modulation of apoptotic and cell cycle pathways. These findings support its potential as a multifunctional therapeutic agent in integrative cancer care.

**Supplementary Information:**

The online version contains supplementary material available at 10.1186/s12906-026-05291-9.

## Introduction

Leukemia represents a diverse group of blood cancers that originate from the uncontrolled growth and impaired differentiation of hematopoietic cells in the bone marrow and peripheral blood [1]. Despite advances in chemotherapy, targeted therapy, and stem cell transplantation, treatment outcomes remain limited by relapse, drug resistance, and systemic toxicity [2].

Moreover, immunosuppression in leukemia patients caused by both the disease and its therapies predisposes them to severe opportunistic infections [3, 4], particularly from multidrug-resistant (MDR) bacteria such as *Streptococcus pyogenes* [4–6]. This Gram-positive bacterium can cause invasive infections, and its growing resistance to antibiotics further complicates treatment [5, 6]. This dual burden of malignancy and infection highlights the urgent need for multifunctional therapeutic agents that can simultaneously target cancer cells and pathogens [4, 7].

Medicinal plants represent a valuable source of such agents, as they contain diverse phytochemicals with proven pharmacological activity [8–10]. Several clinically used chemotherapeutic agents, such as paclitaxel and vincristine, are derived from natural sources [10, 11]. *Punica granatum L*. (p*omegranate*) peel, rich in polyphenols and flavonoids, has been widely recognized for its antioxidant, antimicrobial, and anticancer properties [8, 9, 12]. The rich phytochemical profile of pomegranate makes it a strong candidate for further exploration in oncology and infectious disease research [9, 12].

Prior studies have documented cytotoxic effects of pomegranate extracts against several solid tumors through mechanisms including apoptosis induction, oxidative stress, and cell cycle arrest [13–16]. These antitumor effects are largely attributed to components such as ellagitannins, punicalagins, and flavonoids [9, 12]. However, most of these studies have focused on solid tumors, with far fewer exploring the effects of pomegranate on hematologic malignancies like leukemia [17, 18]. Given the biological differences between solid and blood cancers, further investigation is needed to determine whether similar mechanisms are involved in leukemia cell cytotoxicity [17, 19].

Similarly, antimicrobial activity has been reported against a range of pathogens, including *S. pyogenes*. However, most investigations have been conducted in the context of solid tumors or general antimicrobial screening [7, 20, 21]. Extracts, particularly from the peel and seeds, have been shown to exert bacteriostatic and bactericidal effects against a wide range of pathogens, including *Staphylococcus aureus*, *Escherichia coli*, and *Streptococcus pyogenes* [7, 21, 22]. The mechanisms of action are believed to involve membrane disruption, inhibition of microbial enzymes, and interference with adhesion and biofilm formation [7, 20]. This dual functionality, cytotoxicity against cancer cells and antimicrobial activity against pathogens, positions pomegranate as a promising therapeutic agent, particularly for immunocompromised individuals such as leukemia patients [7, 21, 22].

Despite these encouraging findings, several gaps in the literature remain [10, 23]. Notably, few studies have examined the simultaneous cytotoxic and antimicrobial activities of different pomegranate extracts on leukemia cells and bacterial pathogens [17, 19]. Additionally, the molecular mechanisms underlying *pomegranate’s* effects are not fully understood [12, 23]. Moreover, while crude extracts have shown promise, nano formulation strategies to enhance their bioavailability and efficacy have been underexplored in this context [10, 23, 24]. Furthermore, the comparative effectiveness of different solvent fractions (ethanolic, hexane, aqueous) against both leukemia cells and bacterial strains has not been systematically assessed [16, 20]. Identifying the most bioactive extract and its underlying phytochemicals could pave the way for novel dual-purpose therapeutics [10, 23].

Considering the heightened infection risk in immunocompromised leukemia patients, this study aimed to evaluate pomegranate peel extracts as potential multifunctional agents with both cytotoxic and antimicrobial effects. By employing nano-formulation to enhance bioavailability, we further sought to provide molecular insights into their mechanisms of leukemia cell targeting. We further assess the biological effects of the most active extract and its nano-formulated form on leukemia cell apoptosis, cell cycle regulation, and oxidative stress pathways. To the best of our knowledge, this is one of the first studies to integrate dual cytotoxic and antimicrobial evaluation with molecular docking and nano-formulation, thereby underscoring the translational potential of pomegranate peel as a multifunctional agent for immunocompromised leukemia patients. This comprehensive approach will offer valuable insights into the dual therapeutic potential of pomegranate and its possible application in integrative cancer care [10, 11].

## Materials & methods

### Preparation of pomegranate extracts

The plant material used in this study was *Punica granatum L*. cv. Assiuty, a well-known local Egyptian cultivar. Fresh fruits were purchased from commercial markets in Assiut and New Valley governorates, the primary cultivation regions of this cultivar in Egypt. As the material was not collected from wild populations but sourced from cultivated, commercially available produce, no formal botanical identification or voucher specimen deposition was performed. The identification was based on macroscopic and morphological characteristics consistent with standard botanical references (https://lifetechindia.com/botanical-reference-standards.php).

The fruits were washed thoroughly with distilled water, and the peels were manually separated, shade-dried at room temperature for 10 days, and then finely powdered using an electric grinder. A total of 100 g of the dried peel powder was used for successive extraction using solvents of increasing polarity: hexane (Hex), petroleum ether (PE), ethyl acetate (EA), butanol (BuOH), ethanol (EtOH), and distilled water (Aq). Each extraction was performed by macerating the powder in 500 mL of the respective solvent (peel-to-solvent ratio of 1:5 w/v) at room temperature for 72 h with intermittent stirring. The mixtures were filtered using Whatman No. 1 filter paper, and the filtrates were concentrated under reduced pressure using a rotary evaporator at 45 °C. The aqueous extract was lyophilized to dryness.

The dried crude extracts were weighed, and the extraction yields were calculated as a percentage of the original plant material. The approximate yields were as follows: hexane (2.3%), petroleum ether (2.9%), ethyl acetate (3.6%), butanol (4.8%), ethanol (9.1%), and aqueous extract (6.5%). All extracts were stored in amber glass bottles at − 20 °C until further use in biological and analytical assays to preserve their chemical integrity [25].

For quality control and preliminary profiling, all extracts were subjected to standard phytochemical screening to detect the presence of major secondary metabolites, including alkaloids, flavonoids, tannins, saponins, terpenoids, and phenolics, using conventional qualitative methods.

### Phytochemical compounds analysis by LC-mass QTOF

To identify the bioactive phytochemical constituents present in the pomegranate extracts, particularly the ethanolic extract, liquid chromatography-mass spectrometry quadrupole time-of-flight (LC-MS QTOF) analysis was performed. For sample preparation, 10 mg of the dried ethanolic extract was dissolved in 1 mL of HPLC-grade methanol, vortexed thoroughly, and filtered through a 0.22 μm PTFE syringe filter. The filtrate was transferred into amber LC vials for analysis. The analysis was carried out using an Agilent 1290 Infinity II UHPLC system coupled with an Agilent 6545 QTOF mass spectrometer equipped with an electrospray ionization (ESI) source. Chromatographic separation was achieved using an Agilent ZORBAX Eclipse Plus C18 column (2.1 × 100 mm, 1.8 μm) maintained at 30 °C. The mobile phase consisted of solvent A: 0.1% formic acid in water, and solvent B: 0.1% formic acid in acetonitrile, delivered at a flow rate of 0.3 mL/min using the following gradient: 0–5 min, 5% B; 5–20 min, 5–95% B; 20–25 min, 95% B; 25–30 min, 5% B for re-equilibration. The injection volume was 5 µL [26].

Mass spectrometric detection was performed in both positive and negative ion modes under the following conditions: capillary voltage 3500 V, nebulizer pressure 35 psi, drying gas temperature 300 °C, drying gas flow rate 8 L/min, and fragment or voltage 120 V. The mass range was set from m/z 100 to 1200. Data acquisition and processing were conducted using Agilent MassHunter Workstation software. Accurate mass measurements were ensured by continuous infusion of a reference mass solution throughout the run. The obtained MS and MS/MS spectra were analyzed and matched against online databases, including METLIN, MassBank, and the Human Metabolome Database (HMDB), to tentatively identify the bioactive compounds based on molecular weight, fragmentation patterns, and isotope distributions [27]. Compounds such as ellagic acid, punicalagin, gallic acid, and various flavonoid derivatives were tentatively identified, providing a chemical basis for the observed cytotoxic and antimicrobial activities of the extract.

### Molecular docking

Following the LC-MS QTOF analysis of the ethanolic extract of *Punica granatum*, several phytochemicals with known or predicted biological activities were tentatively identified based on their accurate mass, retention time, and fragmentation patterns. Among these, key bioactive compounds such as ellagic acid, punicalagin, gallic acid, luteolin, kaempferol, and quercetin derivatives were prioritized for further investigation through molecular docking studies. The selection criteria included (1) high relative abundance in the chromatogram, (2) reported cytotoxic or antimicrobial activity in previous literature, and (3) structural features known to interact with apoptosis- and proliferation-related proteins.

Molecular docking was performed to assess the potential affinity of the tested compounds against target proteins. the tested compounds twards to were docked against targets FLT-3, MEK1, and Pi3k (PDB codes: 6jqr, 4u81, and 3v3v).

First, water molecules and unnecessary molecules were removed from the protein complexes. Then, crystallographic disorders and unfilled valence atoms were corrected. The protein structures’ energies were minimized and saved as PDBQT files. The 2D structure of each compound was drawn using Chem-Bio Draw Ultra16.0, saved as an SDF file, and then converted to a 3D structure. Protonation and energy minimization were carried out and saved as PDBQTP files; the prepared ligands were screened against the previous targets, then the best-scoring candidates were selected to dock into the target protein. The processes were conducted using Autodock Vina 1.5.7 software. The docking was conducted using a rigid technique; in this method, the receptor was held rigid while the ligands were allowed to be flexible. During the docking refinement, each molecule was allowed to generate twenty different poses. The docking scores (affinity energy) of the best-fitted poses with the active sites were recorded, and 3D and 2D figures were generated using Discovery Studio 2024 visualizer [28].

### Antimicrobial activity assessment

The antimicrobial activity of the various *Punica granatum* peel extracts was evaluated against *Streptococcus pyogenes* [ATCC 19615], using the agar well diffusion method. The bacterial strain was cultured in Brain Heart Infusion (BHI) broth and incubated overnight at 37 °C to achieve mid-log phase growth. The bacterial suspension was adjusted to a turbidity equivalent to 0.5 McFarland standard (1.5 × 10⁸ CFU/mL). Sterile Mueller-Hinton agar plates were inoculated by evenly spreading 100 µL of the bacterial suspension over the entire agar surface using a sterile swab. Wells of 6 mm diameter were punched into the agar using a sterile cork borer, and each well was filled with 100 µL of the test extract solution (100 mg/mL in DMSO). A well containing DMSO alone served as the negative control, while a standard antibiotic (ampicillin, 10 µg/mL) was used as the positive control.

The plates were incubated at 37 °C for 24 h, after which the antimicrobial activity was assessed by measuring the diameter of the inhibition zones (including the well diameter) around each well using a digital caliper. Each experiment was performed in triplicate, and the results were expressed as the mean ± standard deviation (SD). Extracts showing a zone of inhibition ≥ 12 mm were considered to possess significant antimicrobial activity [29].

### Preparation of nano-formulation of pomegranate peel ethyl acetate extract

The nano-formulation of the ethyl acetate extract of *Punica granatum* peel (PPE-EA) was prepared using a spontaneous emulsification-ultrasonication method with Tween 80 as a stabilizing surfactant. Briefly, 100 mg of the dried ethyl acetate extract was dissolved in 5 mL of absolute ethanol to form the organic phase. Separately, 20 mL of distilled water containing 1% (v/v) Tween 80 was prepared as the aqueous phase. The organic phase was added dropwise into the aqueous phase under continuous magnetic stirring at 1000 rpm for 15 min at room temperature to form a coarse emulsion. This emulsion was then subjected to ultrasonication using a probe sonicator (20 kHz, 100 W) for 10 min with 30-second pulses and 10-second rest intervals to reduce particle size and ensure uniform nano-dispersion. The resulting nano-formulation was cooled to room temperature and stored in amber glass vials at 4 °C until further use [30]. The formulation was visually inspected for clarity and stability, and its particle size distribution and zeta potential were later characterized using dynamic light scattering (DLS) to confirm successful nano-formulation.

The pomegranate peel nano-extract was characterized for particle size and surface charge using Dynamic and Electrophoretic Light Scattering with a Zetasizer Nano ZS. A 2 mg sample was re-dispersed in water, sonicated, and analyzed at 25 °C. Particle size values between 50 and 200 nm confirmed nanoscale formulation, while zeta potential values above ± 30 mV indicated good stability due to electrostatic repulsion (k) [31].

### Cytotoxicity screening and IC₅₀ determination on THP-1 cells

The cytotoxic effects of five different *Punica granatum* peel extracts, “ethanolic, hexane, petroleum ether, ethyl acetate, and butanol,” were evaluated against the human monocytic leukemia cell line THP-1, ATCC ID: TIB-202, using the MTT assay. THP-1 cells were cultured in RPMI-1640 medium supplemented with 10% fetal bovine serum (FBS) and 1% penicillin-streptomycin, maintained at 37 °C in a humidified 5% CO₂ incubator. Cells were seeded in 96-well plates at a density of 1 × 10⁴ cells/well and treated with each extract at a final concentration of 100 µg/mL. After 48 h of incubation, 10 µL of MTT reagent (5 mg/mL) was added to each well and incubated for an additional 4 h. The resulting formazan crystals were dissolved in DMSO, and absorbance was measured at 570 nm using a microplate reader. Cell viability was expressed as a percentage relative to untreated control wells. Among the tested extracts, the ethanolic extract (PPE-EA) showed the highest cytotoxic effect, indicated by the lowest cell viability, and was selected for further evaluation. To enhance its bioactivity and stability, a nano formulation of PPE-EA was prepared using the nanoprecipitation method.

For IC₅₀ determination, THP-1 cells were treated with increasing concentrations (0–100 µg/mL) of both crude extract (PPE-EA) and its nano-formulated counterpart (n-PPE-EA) for 48 h under the same conditions as described above. The IC₅₀ values, the concentration required to reduce cell viability by 50% were calculated from dose–response curves generated using nonlinear regression analysis in GraphPad Prism version 9. All experiments were conducted in triplicate, and results were reported as mean ± standard deviation (SD). A lower IC₅₀ for the nano-formulated extract compared to the crude extract indicated enhanced cytotoxic potency due to nanoencapsulation [32].

### Apoptosis and cell cycle analysis by flow cytometry

Apoptosis was assessed in THP-1 cells using the *Annexin V-FITC/Propidium Iodide (PI) Apoptosis Detection Kit* (*ThermoScientific*,* USA*) following the manufacturer’s protocol. Briefly, THP-1 cells were seeded in 6-well plates at a density of 2 × 10⁵ cells/well and treated with either the PPR-EA, n-PPE-EA, or control (untreated) for 48 h. After treatment, the cells were harvested, washed twice with cold phosphate-buffered saline (PBS), and resuspended in 100 µL of 1X binding buffer. Subsequently, 5 µL of Annexin V-FITC and 5 µL of PI were added to each sample, gently mixed, and incubated in the dark at room temperature for 15 min. After incubation, 400 µL of binding buffer was added, and samples were immediately analyzed by flow cytometry (*Navios EX*,* Beckman Coulter*,* USA*). A total of 10,000 events per sample were acquired. Data were analyzed using quadrant gating to distinguish between viable cells (Annexin V⁻/PI⁻), early apoptotic (Annexin V⁺/PI⁻), late apoptotic (Annexin V⁺/PI⁺), and necrotic cells (Annexin V⁻/PI⁺) [33].

For cell cycle analysis, THP-1 cells were treated under the same conditions and stained using the *Vybrant DyeCycle Violet Stain* (*Invitrogen*,* USA*). After 48 h of treatment, cells were washed with PBS and incubated with 5 µM DyeCycle Violet in pre-warmed culture medium for 30 min at 37 °C, protected from light. Following incubation, the stained cells were analyzed by flow cytometry using the violet laser (excitation/emission: 405/440–460 nm). Cell cycle distribution was assessed using the *Navios EX*,* Beckman Coulter*,* USA software (Beckman Coulter*,* USA*) to quantify the percentage of cells in G₀/G₁, S, and G₂/M phases [34]. All experiments were performed in triplicate, and results were expressed as mean ± standard deviation (SD).

### Oxidative stress and antioxidant activity assays

To evaluate the oxidative stress status and antioxidant defense response in THP-1 cells following treatment with *Punica granatum* extracts, malondialdehyde (MDA) levels and catalase enzyme activity were measured using commercial assay kits from Elabscience (*Elabscience Biotechnology Inc.*,* USA*), following the manufacturer’s protocols. THP-1 cells were treated with the crude ethanolic extract, its nano-formulation, or left untreated (control) for 48 h. After treatment, cells were harvested, washed twice with cold PBS, and lysed using the extraction buffer provided in the kits. Cell lysates were centrifuged at 12,000 rpm for 10 min at 4 °C, and the supernatants were collected for biochemical analysis.

MDA levels, indicative of lipid peroxidation and oxidative membrane damage, were determined using the thiobarbituric acid reactive substances (TBARS) assay kit (Cat. No. E-EL-0060). Absorbance was measured at 532 nm using a microplate reader, and MDA concentration was calculated based on the standard curve provided in the kit. Results were expressed as nmol/mg protein [35].

Catalase activity, a key antioxidant defense enzyme, was assessed using the Catalase Activity Assay Kit (Cat. No. E-BC-K097-M). The assay is based on the decomposition of hydrogen peroxide by catalase, and residual H₂O₂ reacts with a chromogenic substrate to yield a colorimetric product. Absorbance was measured at 405 nm, and enzyme activity was expressed as U/mg protein [36]. Total protein concentration in cell lysates was determined using the Bradford method to normalize the data. All assays were performed in triplicate, and results were expressed as mean ± SD.

### Gene expression analysis

Quantitative gene expression analysis was conducted to investigate the modulatory effects of *Punica granatum* extracts on apoptosis and cell signaling in THP-1 cells. The BCL2, PI3K, and CDK8 genes were selected for expression analysis based on preliminary in silico docking results, which indicated strong binding interactions of pomegranate peel bioactive compounds with these targets. Given their central roles in apoptosis regulation, cell proliferation, and transcriptional control in leukemia, these genes were prioritized to validate the mechanistic predictions at the cellular level. Total RNA was extracted from treated and untreated THP-1 cells using the *RNeasy Mini Kit* (Qiagen, Cat. No. 74104) according to the manufacturer’s protocol. Cells were lysed in RLT buffer containing β-mercaptoethanol, homogenized, and processed through silica-membrane spin columns. RNA purity and concentration were assessed using a NanoDrop spectrophotometer based on the A260/A280 ratio.

cDNA synthesis was performed using the *QuantiTect Reverse Transcription Kit* (Qiagen, Cat. No. 205311), which includes a genomic DNA wipeout step to remove residual DNA. Briefly, 1 µg of total RNA was used in a 20 µL reaction volume following the kit protocol. The reverse transcription reaction was conducted at 42 °C for 15 min, followed by inactivation at 95 °C for 3 min.

Quantitative real-time PCR (qPCR) was performed using *QuantiTect SYBR Green PCR Kit* (Qiagen, Cat. No. 204141) on a *StepOnePlus™ Real-Time PCR System* (Applied Biosystems). Each 20 µL reaction included 10 µL SYBR Green master mix, 2 µL of cDNA template, 1 µL of QuantiTect Primer Assay, and 7 µL of RNase-free water. PCR cycling conditions were as follows: 95 °C for 5 min (initial activation), followed by 40 cycles of 95 °C for 15 s, 60 °C for 30 s, and 72 °C for 30 s. A melt curve analysis was performed at the end of the run to verify amplification specificity. The following Qiagen QuantiTect Primer Assays were used: *BCL2* (Qiagen ID: QT00056174), *PIK3CA* (Qiagen ID: QT00000959), *CDK8* (Qiagen ID: QT00058754), and the *GAPDH* (housekeeping gene, Qiagen ID: QT00079247). All reactions were performed in triplicate, and relative gene expression levels were calculated using the 2^–ΔΔCt method, with GAPDH as the internal control. Data were expressed as mean ± standard deviation (SD) relative to untreated control cells [36].

### Statistical analysis

All experimental data were analyzed using IBM SPSS Statistics version 25.0 (IBM Corp., Armonk, NY, USA) and GraphPad Software, San Diego, CA, USA. Results were expressed as mean ± standard deviation (SD) from at least three independent experiments. Normality of data distribution was assessed using the *Shapiro–Wilk* test. For comparisons between multiple groups, *one-way analysis of variance* (ANOVA) followed by *Tukey’s post hoc test* was applied to determine statistically significant differences. For pairwise comparisons ( IC₅₀ values of crude vs. nano-formulated extracts), an independent samples t-test was used. A *p*-value < 0.05was considered statistically significant. Dose–response curves and IC₅₀ values were generated using nonlinear regression analysis (log\[inhibitor] vs. normalized response–variable slope) in GraphPad Prism. All graphs and bar charts were also generated in GraphPad Prism.

## Results

### Phytochemical profiling and biological interpretation of identified compounds

Table 1 presents the Liquid Chromatography-Mass Spectrometry (LC-MS), Quadrupole Time-of-Flight (QTOF) analysis of the PPE-EA. Results revealed a rich spectrum of bioactive compounds with diverse structural classes, including flavonoids, phenolic acids, tannins, anthocyanins, and phytosterols. A total of 49 compounds were tentatively identified based on their retention times, molecular weights, and spectral fragmentation patterns (supplementary Table 1). Among the major constituents, chlorogenic acid (20,232.81 µg/g), naringenin (6,968.48 µg/g), gallic acid (6,013.80 µg/g), campesterol (13,606.15 µg/g), and quercetin (4,390.18 µg/g) were found in high concentrations, suggesting their potential contribution to the extract’s bioactivity.

**Table 1:** Key bioactive compounds identified by LC-MS QTOF in the ethyl acetate extract of *Punica granatum* peel, along with their chemical classes, reported biological activities

Compound Name	Class	Reported Bioactivities	Conc. U/g
Chlorogenic acid	Phenolic acid	Antioxidant, antibacterial, anti-inflammatory	20232.807
Naringenin	Flavanone	Cytotoxic, pro-apoptotic, anti-inflammatory	6968.481
Gallic acid	Phenolic acid	Antioxidant, antimicrobial, cytotoxic	6013.795
Campesterol	Phytosterol	Anti-inflammatory, immunomodulatory	13606.152
Quercetin	Flavonol	Cytotoxic, apoptotic, cell cycle arrest (PI3K/Akt, BCL2 inhibition)	4390.180
Luteolin	Flavone	Apoptotic, anti-angiogenic, antioxidant	2574.644
Kaempferol-3-O-rutinoside	Flavonol glycoside	Apoptotic, anti-proliferative, antioxidant	883.749
Myricetin	Flavonol	Apoptotic, antioxidant, and cell cycle modulation	158.956
Punicalin	Ellagitannin	Anti-proliferative, DNA protection	311.826
Punicalagin	Ellagitannin	Apoptotic, anti-inflammatory, and antioxidant	155.682
Ellagic acid	Polyphenol	Antioxidant, anti-mutagenic, apoptotic	727.114
Pedunculagin	Ellagitannin	Cytotoxic, DNA protective	101.545
Caffeic acid	Phenolic acid	Antioxidant, antimicrobial, cytotoxic	100.801
Ferulic acid	Phenolic acid	Antioxidant, anti-inflammatory, cytotoxic	317.509
Sinapic acid	Phenolic acid	Antioxidant, antimicrobial, neuroprotective	528.822
Betulinic acid	Triterpenoid	Selective cytotoxicity, mitochondrial apoptosis	235.178
Lupeol	Triterpenoid	Cytotoxic, anti-inflammatory, and apoptosis induction	245.626
Gallocatechin	Flavanol	Antioxidant, anti-metastatic	61.147
Epicatechin gallate	Flavanol	Apoptotic, antioxidant, anti-proliferative	335.378
Gallocatechin gallate	Flavanol	Antioxidant, antimicrobial, cytotoxic	369.346

### Molecular docking

The identified compounds were screened against different targets to assess their possible binding affinity against *FLT-3*, *MEK1*, and *Pi3K.* The results of the top screened compounds are illustrated in Table 2.

**Table 2:** Molecular docking analysis of the tested compounds against the screened target protein (*FLT-3*,* MEK1*,* and PI3K*)

Tested compounds	Compounds	RMSD value (Å)	Docking (Affinity) score (kcal/mol)
*FLT-3*	Compound 19 “*Triacontane*”	0.91	-8.89
	Compound 34 “*Copaene*”	1.46	-8.95
	Compound 36” *Squalene*”	1.55	-8.77
	Co-crystalized ligand	0.65	-8.41
*MEK1*	Compound 19 “*Triacontane*”	1.54	-7.07
	Compound 32” *Epicatechin gallate*”	1.07	-9.03
	Co-crystalized ligand	1.01	-9.78
*Pi3K*	Compound 9 “*Punigluconin*”	1.26	-7.99
	Compound 14 “*Campesterol*”	1.16	-7.70
	Compound 20 “*Apigenin*”	1.36	-7.36
	Co-crystalized ligand	0.91	-7.08

The table presents the root-mean-square deviation (RMSD) values and docking (affinity) scores in kcal/mol for each compound in comparison to their corresponding co-crystallized ligands

#### Molecular docking of target compounds against FLT-3

**Fig. 1:**
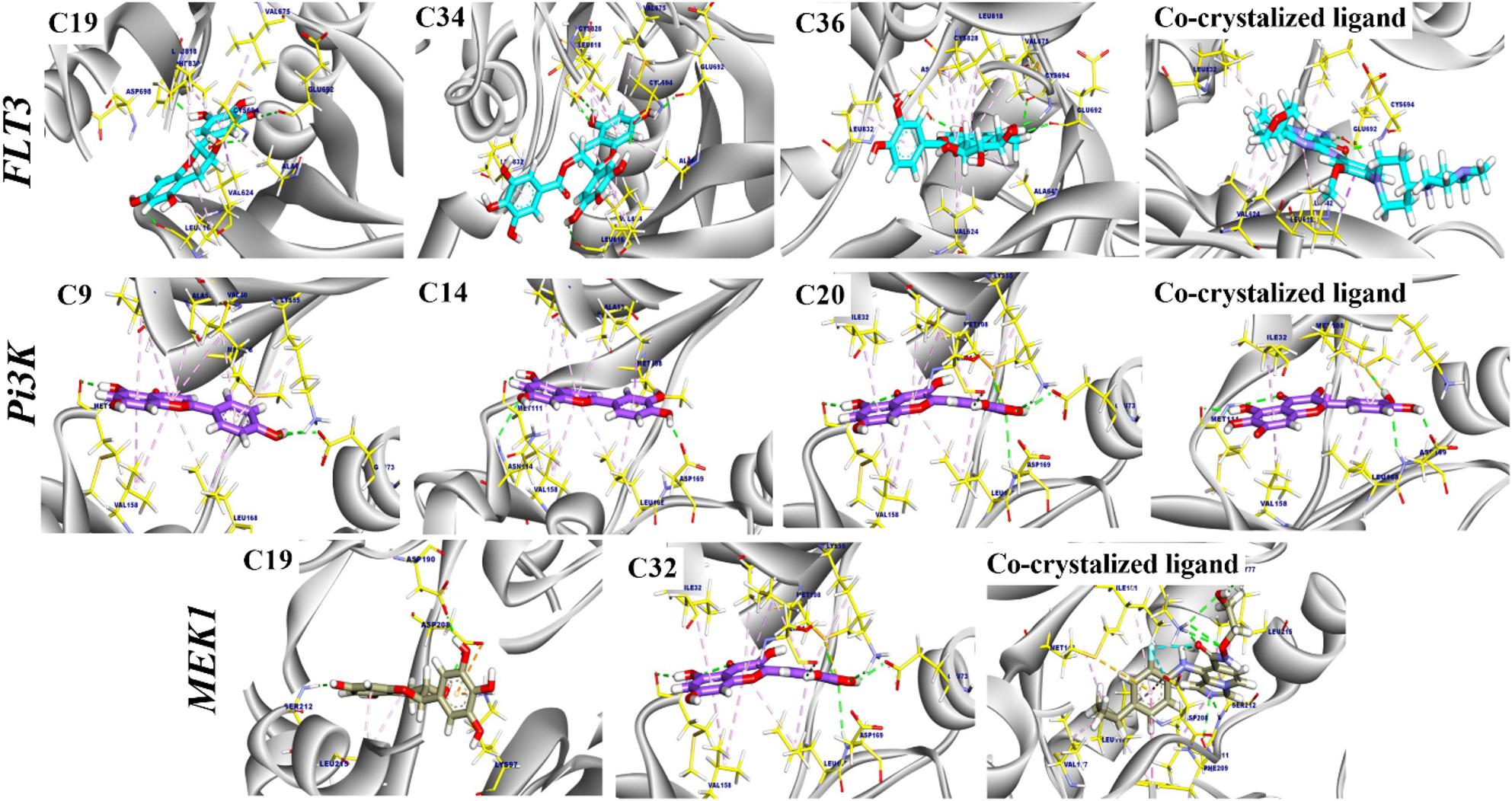
The proposed binding modes of selected compounds docked into the active sites of *FLT-3*,* MEK1*, and *PI3K* proteins. The ligands are shown within the binding pockets of their respective targets, illustrating key interactions and conformations that may underlie their binding affinities

The top tested *Triacontane* (19), *Copaene* (34), and *Squalene* (36) exhibited an affinity score of -8.89, -8.95, -8.77, and − 8.41 kcal/mol, respectively. Triacontane (19) interacted with Ala642, Val675, Val624, Leu616, and Leu818 by seven hydrophobic π-interactions. Moreover, it formed five hydrogen bonds with Leu616, Phe830, Glu692, and Cys694 with distances of 2.71, 2.20, 2.44, 2.25, and 2.39 Å, respectively. Additionally, *Copaene* (34) formed ten hydrophobic π-interactions with Leu832, Leu818, Cys828, Leu616, Val675, Ala642, and Val624. Additionally, the interactions were supported by five hydrogen bonds: Asp829, Cys828, Leu616, Cys694, and Glu692, with distances of 2.91, 2.95, 2.97, 2.05, and 2.17 Å, respectively. Furthermore, Squalene (36) formed eight hydrophobic π-interactions with Val675, Cys828, Val624, Ala642, Leu818, Leu832, and formed four hydrogen bonds with Asp829, Glu692, and Cys694 with distances of 2.442.05, 2.92, 2.01, and 2.36 Å. The co-crystalized ligand complexed with FLT-3 exhibited an affinity score of -8.41 kcal/mol. It formed seven hydrophobic π-interactions with Val624, Ala642, Leu818, Leu832, and Leu616. Furthermore, interacted with Glu692 and Cys694 by two hydrogen bonds with distances of 1.86 and 1.81 Å, respectively (Fig. 1).

#### Molecular docking of target compounds against MEK1

The binding mode of *Triacontane* (19) exhibited a binding energy of -7.07 kcal/ mol. against *MEK1*. four hydrophobic π-interactions were observed with Leu215, Lys97, and Asp208. Additionally, *Triacontane* formed three hydrogen bonds with Asp190, Asp208, and Ser212 with bond lengths of 2.10, 1.99, and 2.14 Å. Moreover, *Epicatechin gallate* (32) exhibited an affinity score of -9.03 kcal/ mol against *MEK1*. It interacted with Leu215, Met143, Cys207, Leu118, Ile141, and Phe209 by six hydrophobic π-interactions. Additionally, seven hydrogen bonds were noted with Met143, Lys97, Gly77, Asp190, Lys192, Ser212, and Val211 with distances of 2.80, 2.52, 1.92, 2.04, 2.34, 2.47, and 3.06. The co-crystalized ligand complexed with *MEK1*( as an inhibitor) exhibited an affinity score of -9.78 kcal/mol, the co-crystalized ligand formed twelve hydrophobic π-interactions with Ile141, Phe209, Leu118, Met143, Val127, Asp208, and Leu215, Furthermore interacted with Gly77, Lys97, Ser212 and Val211 by six hydrogen bonds with distances of 2.24, 2.76, 2.86, 1.78, 1.97, and 2.75 Å (Fig. 1).

#### Molecular docking of target compounds against Pi3K

The binding mode of *Punigluconin* (9) exhibited a binding affinity of -7.99 kcal/ mol. against *Pi3K*. It formed eleven hydrophobic π-interactions with Ile32, Val158, Ala53, Val40, Met108, and Leu168. Moreover, it interacted with Met111, Glu73, and Lys2.56 by five hydrogen bonds with distances of 2.38, 2.60, 2.17, 2.56, and 2.31 Å. The binding mode of *Campesterol* (14) exhibited an affinity score of -7.70 kcal/ mol. against *Pi3K*. it formed nine hydrophobic π-interactions with Met108, Val40, Leu168, Ala53, Val158, and Ile3. Additionally, six hydrogen bonds were oriented with Asn114, Met111, Met108, and Asp169 with distances of 2.83, 2.31, 2.00, 2.21, 2.31, and 1.83 Å. Furthermore, *Apigenin* (20) exhibited an affinity score of -7.36 kcal/ mol. against *Pi3K*. and formed ten hydrophobic π-interactions with Ile32, Met108, Ala53, Val158, Leu168, Val40, and showed eight hydrogen bonds with Ser2.74, Lys2.42, Asp169, Glu73, Met108, and Met111 with distances of 2.74, 2.42, 2.71, 2.18, 2.38, 2.38, 2.53, and 2.23 Å. Additionally, the co-crystalized inhibitor, which complexed with *Pi3K* target site exhibited an affinity score of -7.08 kcal/mol, which formed six hydrophobic π-interactions with Lys55, Val40, Leu168, Val158, Ile32 and Met108, Furthermore interacted with Asp169, Met108, and Met111 by six hydrogen bonds with distances of 1.91, 2.87, 2.35, 2.56, 2.00, and 2.73 Å (Fig. 1).

### Characterization of pomegranate peel ethyl acetate extract nano-formulation

**Fig. 2:**
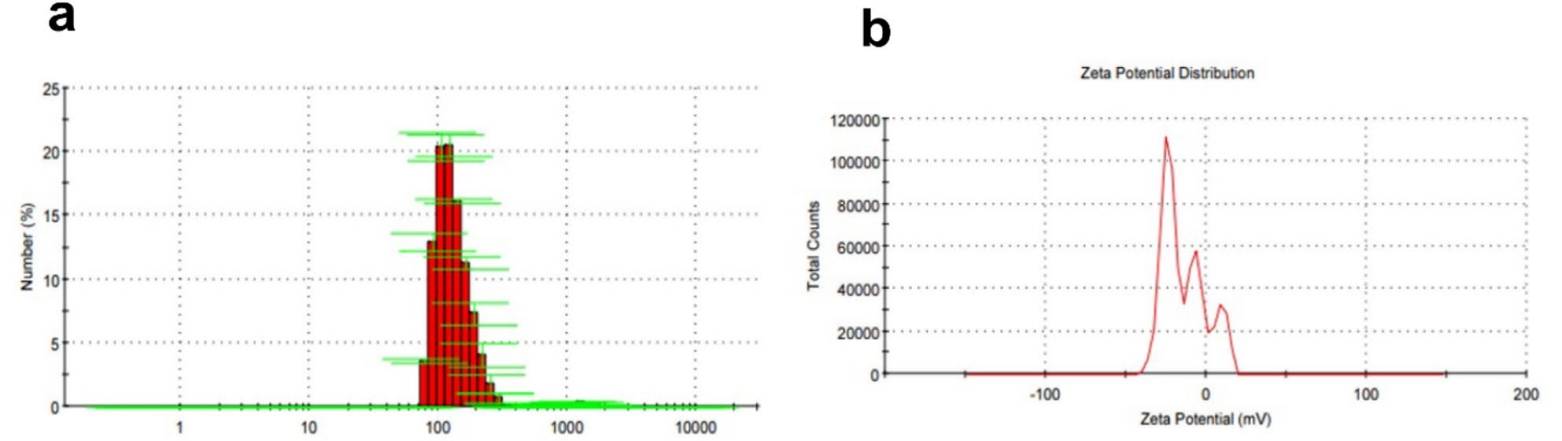
Characterization of pomegranate peel-derived nanoparticles. **a** Dynamic Light Scattering (DLS) analysis shows a narrow and uniform particle size distribution with a peak around 100 nm, indicating monodispersity and nanoscale formulation. **b** Zeta potential measurement reveals an average surface charge of -14.0 mV, suggesting moderate colloidal stability. The distribution curve includes multiple peaks, reflecting surface charge heterogeneity, while the conductivity value (0.0368 mS/cm) indicates a moderate ionic strength of the suspension

The characterization of pomegranate peel-derived nanoparticles revealed a well-controlled and stable formulation. Dynamic Light Scattering (DLS) analysis ( Fig. 2a) showed a narrow size distribution ranging from 70 to 150 nm, with a peak around 100 nm, indicating high monodispersity and uniformity, key features for enhanced bioavailability and predictable drug delivery. The absence of larger aggregates confirmed effective synthesis via emulsification and ultrasonication. Zeta potential measurements averaged − 14.0 mV with some heterogeneity in surface charge distribution (Fig. 2b), suggesting moderate colloidal stability. While suitable for short-term or immediate use, long-term stability may benefit from further optimization. Overall, the formulation demonstrates promising characteristics for pharmaceutical or nutraceutical applications.

### Antibacterial efficacy of *Punica granatum* extracts against *Streptococcus pyogenes* (Table 3; Fig. 3)

The antibacterial activities of various *Punica granatum* peel extracts prepared with solvents of increasing polarity were assessed against *Streptococcus pyogenes* using the agar diffusion assay. The ethyl acetate extract displayed the most pronounced activity among all crude extracts, with a significant zone of inhibition measuring 10.5 ± 1.1 mm (*p* < 0.0001), classifying it as a strong antimicrobial agent. In contrast, non-polar extracts such as hexane and petroleum ether exhibited weak antibacterial activity. Moderately polar solvents like butanol and ethanol produced intermediate zones, while the aqueous extract showed a mild effect, indicating that mid-polar solvents are more effective in extracting antibacterial phytoconstituents from pomegranate peel.

To further assess the potency of the ethyl acetate extract, its effect was examined at the IC₅₀ concentration determined on THP1 cells (1.48 µg/mL) and compared to its nano-formulated version at a significantly lower concentration (0.19 µg/mL). Remarkably, the crude extract at IC₅₀ exhibited a stronger zone of inhibition (12.5 ± 0.7 mm) than the crude form, while the nano-formulated extract produced the highest antimicrobial effect (15.6 ± 0.5 mm), demonstrating both statistical significance over the DMSO control and over the non-nanoformulated ethyl acetate extract (*p* < 0.05). These findings suggest that nanoencapsulation enhances the delivery and bioavailability of active constituents, allowing for superior antibacterial efficacy at substantially reduced concentrations.

**Table 3:** Zone of Inhibition (mm) for various solvent-based extracts of *Punica granatum* peel and ethyl acetate extract formulations against *Streptococcus pyogenes*

No	Extract	Zone of inhibition (mm)	Interpretation	*p*-value
1	DMSO	0.82 ± 0.11	No reaction	
2	Hexane	4.5 ± 0.6	Weak	0.0001^a^
3	Petroleum Ether	5.2 ± 0.4	Weak	0.0001^a^
4	Ethyl Acetate	10.5 ± 1.1	Strong	0.0001^a^
5	Butanol	6.8 ± 0.9	Moderate	0.0001^a^
6	Ethanol	6.2 ± 0.7	Moderate	0.0001^a^
7	Aqueous	5.5 ± 0.5	Mild	0.0001^a^
8	PPE-EA (1.48 µg/mL)	12.5 ± 0.7	Strong	0.0001^a^
9	n-PPE-EA (0.19 µg/mL)	15.6 ± 0.5	Strong	0.000

Comparative analysis was conducted using one-way ANOVA followed by Tukey’s post hoc test

Values represent mean ± SD (*n* = 3)

*PPE-EA* Ethyl acetate fraction of pomegranate peel extracts, *n-PPE-EA* Nano-formulated ethyl acetate fraction of pomegranate peel extracts, *DMSO* Dimethyl sulfoxide

^a^Statistical significance compared to the Negative control (DMSO) (*p* < 0.05)

^b^Statistical significance compared to the PPE-EA (1.48 µg/mL) (*p* < 0.05)

**Fig. 3:**
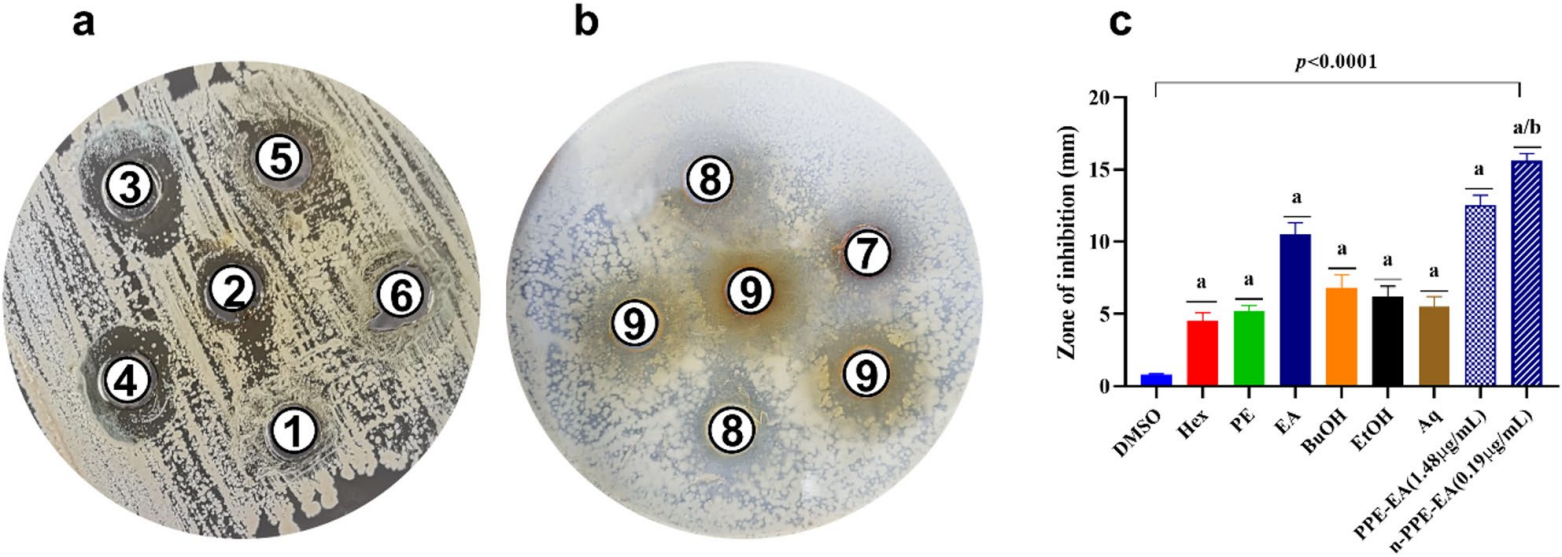
Comparative antibacterial activity of *Punica granatum extracts* (**a**), ethyl acetate extract and its nano formulation (**b**) against *Streptococcus pyogenes.* Bar chat presents the Zone inhibition for each extract versus DMSO **c**. The nano-formulated extract exhibited significantly higher inhibition at a lower concentration compared to both the native extract and all other solvent extracts tested. Values represent mean ± SD (*n* = 3). Comparative analysis was conducted using one-way ANOVA followed by Tukey’s post hoc test. ^a^:statistical significance compared to THP1 cells cultured in DMEM media (*p* < 0.05), ^b^:statistical significance compared to THP1 cells cultured in DMEM media supplemented with 1.48 µg/mL of PPE-EA (*p* < 0.05)

### Cytotoxic effects and potency enhancement of pomegranate peel extracts against THP-1 cells

**Fig. 4:**
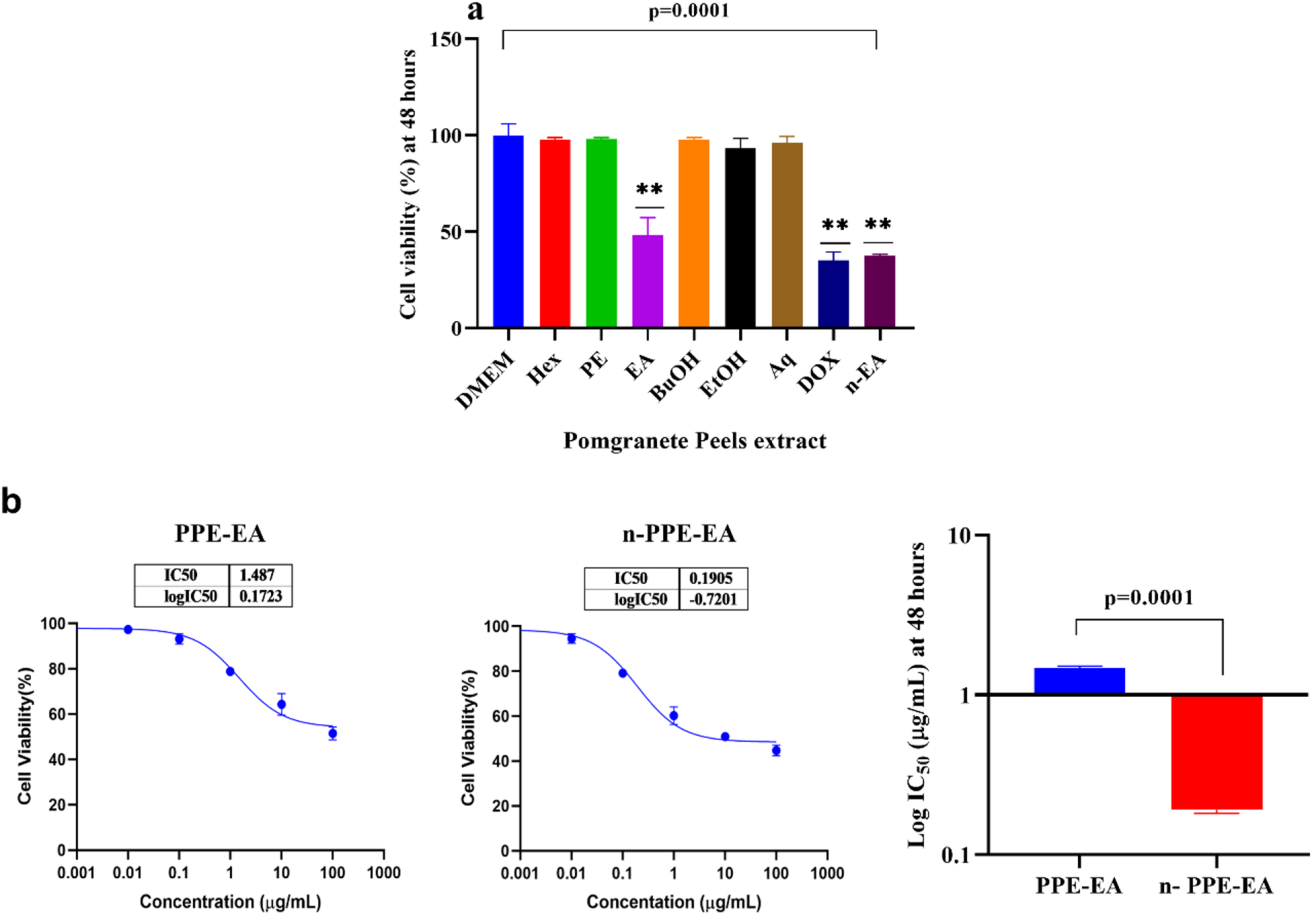
**a** Average percentage of THP-1 cell viability after treatment with six different pomegranate peel extracts (PPE-Hex, PPE-PE, PPE-EA, PPE-BuOH, PPE-EtOH, and PPE-Aq) at a fixed concentration for 48 h. Cell viability was measured using the MTT assay. The ethyl acetate extract (PPE-EA) showed a significant reduction in viability, like the standard chemotherapeutic agent doxorubicin (DOX). Data are presented as mean ± SD; *p* < 0.0001 compared to DMEM. **b** IC₅₀ values of the ethanolic extract (PPE-EA) in crude versus nano-formulated form, determined from serial dilution experiments and calculated by non-linear regression analysis of viability data, with a Bar chart comparing log IC₅₀ values between the crude ethyl acetate extract (PPE-EA) and its nano-formulated counterpart (n-PPE-EA) on THP-1 cells after 48 h of exposure. Nano formulation significantly enhanced cytotoxic potency, reducing the IC₅₀ value from 1.48 µg/mL to 0.191 µg/mL. Statistical significance was determined using an unpaired t-test; *p* = 0.0001

The cytotoxic potential of six different pomegranate peel extracts (PPE) was assessed against THP-1 leukemia cells using the MTT assay (Table 4; Fig. 4a). Among the tested solvent fractions, PPE-Hex, PPE-PE, PPE-EA, PPE-BuOH, PPE-EtOH, and PPE-Aq, PPE-EA exhibited the exhibited the strongest combined cytotoxic and antimicrobial activities and was therefore selected for Nano formulation and further mechanistic analysis. PPE-EA showed a significant reduction in percentage viability, compared to untreated cells (*p* < 0.0001). In contrast, the other extracts (Hex, PE, BuOH, EtOH, and Aq) maintained cell viabilities above 90%, indicating minimal cytotoxic effects at the tested concentrations. Dunnett’s multiple comparison test confirmed that only PPE-EA and DOX showed statistically significant reductions in viability compared to DMEM.

**Table 4:** Percentage of THP-1 cell viability following 48-hour treatment with pomegranate peel extracts (PPE) prepared using different solvents

Group	Cell viability (%)	*P*-value	IC_50_	*P*-value
DMEM	99.9 ± 6.06	-		-
Hexane (Hex)	97.7 ± 1.05	0.99		
Petroleum ether (PE)	98.0 ± 0.79	0.96		
Butanol (BuOH)	97.8 ± 1.06	0.99		
Ethanol EtOH)	93.3 ± 5.09	0.421		
Aqueous (Aq)	96.03 ± 3.5	0.848		
Doxorubicin (DOX)	35.2 ± 4.32	0.0001		
Ethyl acetate (PPE-EA)	48.2 ± 9.2	0.0001	1.48 ± 0.032	
Nano-PPE-EA	37.42 ± 1.8	0.0001	0.191 ± 0.01	0.0001

*DMEM* Dulbecco’s Modified Eagle Medium. Values are presented in mean ± SD. The *p*-value is calculated compared to the DMEM group, “untreated cells”, Ethyl acetate *EA* Ethyl acetate extract of Pomegranate, *Nano-PPE-EA* Nanoform of Ethyl acetate extract of Pomegranate

Among the tested fractions, the ethyl acetate extract (PPE-EA) exhibited the strongest combined cytotoxic and antimicrobial activities and was therefore selected for Nano formulation and further mechanistic analysis. To further quantify the potency of the PPE-EA extract, the half-maximal inhibitory concentration (IC₅₀) was calculated (Fig. 4b). The crude PPE-EA exhibited an IC₅₀ value of 1.48 ± 0.032 µg/mL, reflecting high cytotoxic efficacy. Upon nano formulation (n-PPE-EA), the cytotoxic potency was significantly enhanced, with a reduced IC₅₀ of 0.191 ± 0.01 µg/mL (*p* = 0.0001). This nearly eightfold improvement in potency is attributed to the increased bioavailability, cellular uptake, and stability of the phytochemicals in the nanoformulated form.

### Cytotoxic effects of PPE-EA and n-PPE-EA on apoptosis and cell cycle progression in THP1 cells

**Fig. 5:**
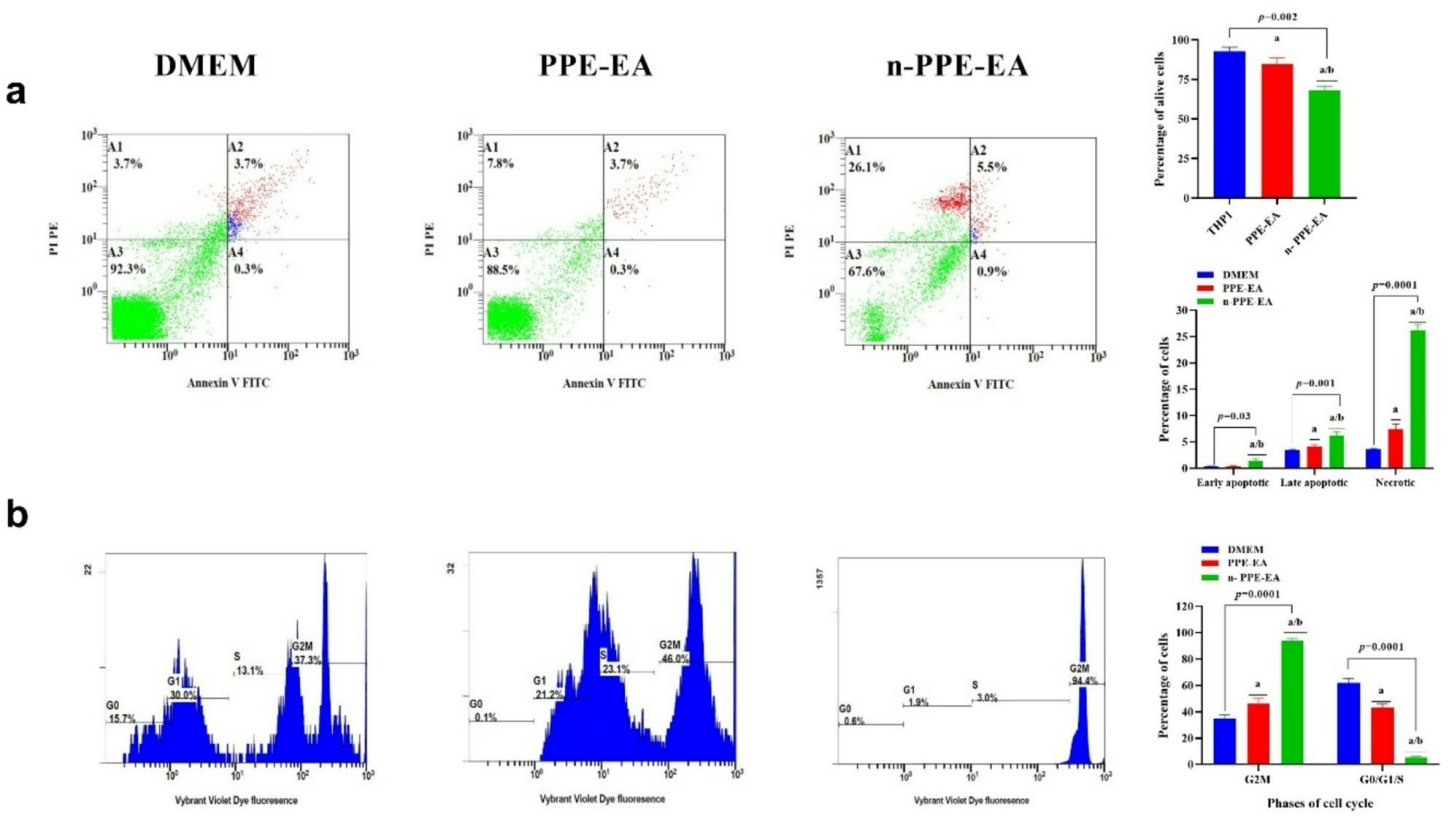
Flow cytometry analysis of (**a**) apoptosis and (**b**) cell cycle distribution in THP-1 cells treated with PPE-EA and n-PPE-EA. Annexin V–FITC/PI dual staining identified four cell populations: A3 = alive cells (Annexin V–/PI–), A4 = early apoptotic cells (Annexin V+/PI–), A2 = Late apoptotic cells (Annexin V+/PI+), A1 = necrotic cells (Annexin V-/PI+). The bar chart summarizes the alive population (A3) and the early apoptotic (A4), late apoptotic (A2), and necrotic (A1) in the three tested groups. Data show increased apoptosis and G2M arrest with nano-formulation. Comparative analysis was conducted using one-way ANOVA followed by Tukey’s post hoc test. ^a^: statistical significance compared to THP-1 cells cultured in DMEM (*p* < 0.05), ^b^: statistical significance compared to THP-1 cells cultured in DMEM supplemented with 1.48 µg/mL of PPE-EA (*p* < 0.05). Data are presented in mean ± SD. Abbreviations: PPE-EA: ethyl acetate extract from *Punica granatum*, n-PPE-EA: ethyl acetate extract from *Punica granatum* in its nano formulation. DMEM: Dulbecco’s Modified Eagle Medium

Table 5; Fig. 5 demonstrated that both PPE-EA and its nano-formulated version (n-PPE-EA) induce statistically significant but differential changes in cell fate distribution activity and cell cycle dynamics in THP1 leukemia cells,, with n-PPA-EA exhibiting a more pronounced effect. As shown in Fig. 5a, the most pronounced change was observed in the necrotic cell population, which increased to 26% in n-PPE-EA–treated cells, compared to 7.6% in PPE-EA–treated cells and 3.7% in DMEM control cells (*p* < 0.0001). Late apoptotic cells also showed a significant increase following PPE-EA and n-PPE-EA treatment, whereas early apoptotic cells exhibited only a mild but statistically significant increase in the n-PPE-EA group compared with DMEM and PPE-EA groups (*p* = 0.03).These changes were accompanied by a corresponding reduction in the alive cell population, particularly in cells treated with the nano-formulated extract, indicating that n-PPE-EA predominantly shifts cell fate toward non-viable states, with necrosis representing the dominant mode of cell death rather than classical early apoptosis. This was accompanied by a significant reduction in viable cells, particularly with the nano-formulated extract. Cell cycle analysis (Fig. 5b) revealed a profound shift to G2/M phase arrest in treated groups. PPE-EA induced an increase in G2M phase cells, while n-PPE-EA caused a further elevation (*p* < 0.0001), indicating effective disruption of cell cycle progression.

**Table 5:** Apoptotic, cell cycle responses, oncogenic genes (*BCL2*, *PI3K*, *CDK8*)expression, and oxidative stress markers (catalase, MDA) of THP1 cells after treatment with PPE-EA and its nano-formulated derivative (n-PPE-EA) compared to THP1 cells cultured in DMEM

Group	DMEM	PPE-EA	*n*-PPE-EA	*p*-value
Apoptosis by Annexin V/PI				
* Alive cells (Annexin V-/PI-)*	92.9 ± 2.40	84.8 ± 3.97^a^	67.9 ± 2.87^a/b^	0.002
* Early apoptotic (Annexin V+/PI-)*	0.3 ± 0.05	0.3 ± 0.01	0.9 ± 0.02^a/b^	0.03
* Late apoptotic (Annexin V+/PI+)*	3.7 ± 0.92	3.7 ± 0.51^a^	5.5 ± 1.12^a/b^	0.001
* Necrotic (Annexin V-/PI+)*	3.7 ± 0.12	7.4 ± 0.25^a^	26.1 ± 3.2^a/b^	0.0001
Cell cycle assay				
* G1/S phase*	61.7 ± 3.65	43.3 ± 2.72^a^	5.40 ± 0.62 ^a/b^	0.0001
* G2M*	34.5 ± 3.43	46.1 ± 4.05^a^	93.8 ± 1.83 ^a/b^	0.0001
Gene expression (log_10)_				
* BCL2*	1.00 ± 0.11	0.56 ± 0.15^a^	0.21 ± 0.05^a/b^	0.0004
* PI3K*	1.03 ± 0.09	0.78 ± 0.08	0.44 ± 0.05^a^	0.018
* CDK8*	1.06 ± 0.05	0.29 ± 0.043^a^	0.04 ± 0.01^a^	0.0039
Oxidative markers				
* Catalase (µmol/mg protein)*	8.94 ± 2.03	14.8 ± 1.95^a^	21.9 ± 4.34^a^	0.005
* MDA (nM/mg protein)*	32.6 ± 2.43	21.5 ± 0.84	14.5 ± 1.88^a/b^	0.0001

Data are presented in mean ± SD. Comparative analysis was conducted using one-way ANOVA followed by Tukey’s post hoc test

*Abbreviations*: *PPE-EA* Ethyl acetate extract from *Punica granatum*, *n-PPE-EA* Ethyl acetate extract from *Punica granatum* in its nano formulation, *DMEM* Dulbecco’s Modified Eagle Medium, *BCL2* *B-cell Lymphoma-2 gene*, *PI3K* *Phospho-inositol 3 kinase gene*, *CDK8* *Cyclin-dependent kinases 8 gene*, *MDA* Malonaldehyde (Lipid peroxidase)

^a^Statistical significance compared to THP1 cells cultured in DMEM media (*p* < 0.05)

^b^Statistical significance compared to THP1 cells cultured in DMEM media supplemented with 1.48 µg/mL of PPE-EA (*p* < 0.05)

### Nano-PPE-EA enhances antioxidant activity in THP1 cells

**Fig. 6:**
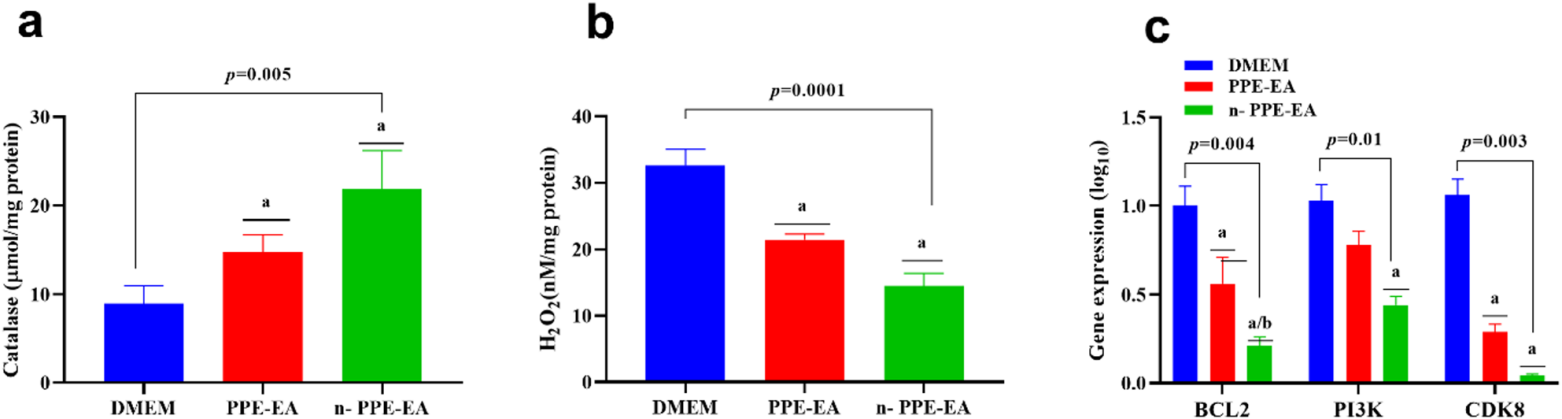
Graphical representation of oxidative stress parameters [catalase (**a**) and MDA (**b**)] and gene expression (BCL2, PI3K, CDK8) (**c**) in THP1 cells after treatment with PPE-EA and n-PPE-EA. Nano-formulated extract significantly enhanced antioxidant capacity and downregulated oncogenic gene expression. Values are presented in mean and standard deviation. Comparative analysis was conducted using one-way ANOVA followed by Tukey’s post hoc test. ^a^: statistical significance compared to THP1 cells cultured in DMEM media (*p* < 0.05), ^b^: statistical significance compared to THP1 cells cultured in DMEM media supplemented with 1.48 µg/mL of PPE-EA(*p* < 0.05). Abbreviations: PPE-EA: ethyl acetate extract from *Punica granatum*, n-PPE-EA: ethyl acetate extract from *Punica granatum* in its nano formulation. DMEM: Dulbecco’s Modified Eagle Medium, *BCL2*: *B-cell Lymphoma-2 gene*, *PI3K*: *Phospho-inositol 3 kinase gene*, *CDK8*: *Cyclin-dependent kinases 8 gene*, MDA: Malonaldehyde (Lipid peroxidase)

As shown in Table 5; Fig. 6a, and Fig. 6b, the antioxidant profile of THP1 cells was significantly improved following treatment. Catalase activity, an indicator of enzymatic antioxidant defense, increased from 8.94 µmol/mg protein in control cells to 14.8 µmol/mg with PPE-EA and further to 21.9 µmol/mg with n-PPE-EA (*p* = 0.005). In parallel, levels of malondialdehyde (MDA), a lipid peroxidation marker reflecting oxidative stress, decreased markedly from 32.6 nM/mg protein in the control group to 21.5 nM/mg protein with PPE-EA and 14.5 nM/mg protein with n-PPE-EA (*p* = 0.0001).

### Nano-PPE-EA enhances and suppresses oncogenic gene expression in THP1 cells

Treatment with PPE-EA and n-PPE-EA led to a significant downregulation of oncogenic gene expression in THP1 cells. The anti-apoptotic gene *BCL2* decreased from 1.00 (log₁₀ scale) in the control (DMEM) group to 0.56 with PPE-EA, and further to 0.21 with the nano-formulation (*p* = 0.0004). Similarly, *PI3K* expression dropped from 1.03 to 0.78 with PPE-EA and to 0.44 with n-PPE-EA (*p* = 0.018), while *CDK8* expression was reduced from 1.06 in control to 0.29 and 0.04 with PPE-EA and n-PPE-EA, respectively (*p* = 0.0039). Results are presented in Table 5; Fig. 6c.

## Discussion

This study investigated the dual cytotoxic and antimicrobial activities of *Punica granatum* peel extracts, with a focus on the ethyl acetate fraction (PPE-EA) and its nano-formulated form (n-PPE-EA). Our results demonstrate that n-PPE-EA significantly enhances both anticancer and antibacterial effects, highlighting the potential of nano-formulation strategies to improve the therapeutic profile of natural products. Importantly, this dual activity addresses two critical challenges in leukemia management: malignant cell survival and opportunistic infections in immunocompromised patients. The cytotoxicity assays revealed that n-PPE-EA exerted a markedly lower IC₅₀ compared to crude PPE-EA, consistent with the improved uptake and intracellular delivery often reported with phytochemical nano-formulations. These findings align with previous studies showing that nanostructured delivery systems enhance the bioavailability and potency of pomegranate polyphenols against cancer cells [37]. In parallel, antimicrobial testing against *S. pyogenes* revealed a substantial increase in activity upon nano-encapsulation, with inhibition zone diameters increasing from 10.5 ± 1.1 mm (PPE-EA) to 15.6 ± 0.5 mm (n-PPE-EA) at equivalent concentrations.

Mechanistic investigations further revealed that n-PPE-EA exerted its cytotoxic effect through activation of intrinsic apoptotic pathways. Apoptosis assays showed that the predominance of necrotic cell death observed with n-PPE-EA treatment suggests that the nano-formulated extract may induce acute cytotoxic stress rather than selectively activating canonical apoptotic pathways. Nanoparticle-mediated delivery is known to enhance cellular uptake, intracellular accumulation, and local concentration of bioactive compounds, which may overwhelm cellular homeostatic mechanisms, leading to membrane disruption, mitochondrial dysfunction, and loss of membrane integrity, hallmarks of necrotic cell death [38]. Moreover, the relatively modest increase in early apoptotic cells, alongside a marked rise in necrosis, indicates that apoptosis may occur transiently or incompletely before rapid progression to secondary necrosis [39]. This pattern has been reported for phytochemical-based nano-formulations, where enhanced bioavailability can shift the balance from regulated apoptosis toward energy-independent or stress-induced necrotic pathways, particularly in highly proliferative leukemia cells [40]. Together, these findings suggest that while PPE-EA and n-PPE-EA modulate apoptotic signaling, the nano-formulated extract primarily exerts its anti-leukemic effect through induction of necrotic cell death, which may contribute to its stronger cytotoxic profile.

At the molecular level, n-PPE-EA downregulated the anti-apoptotic gene *BCL2* and inhibited the PI3K/Akt signaling cascade, a pathway often upregulated in leukemia to promote survival and chemoresistance. This was accompanied by increased activation of caspase-3, confirming the involvement of mitochondrial-mediated apoptosis. Additionally, cell cycle analysis indicated significant G2/M arrest following n-PPE-EA treatment, which was supported by the downregulation of cyclin-dependent kinase 8 (CDK8), a transcriptional regulator that facilitates cell cycle progression and leukemogenesis. These findings collectively demonstrate that n-PPE-EA suppresses leukemia cell proliferation by simultaneously inducing apoptosis and halting cell cycle arrest at the G2M checkpoint.

At the mechanistic level, our observation that n-PPE-EA downregulated BCL2 and PI3K, while inducing caspase activity and G2M arrest via suppression of CDK8, underscores its ability to modulate multiple oncogenic pathways. Ismail et al. [41] reported that nano-formulated PPE significantly suppressed the proliferation of acute myeloid leukemia (AML) cells via downregulation of the PI3K/Akt pathway and inhibition of *BCL2*, alongside increased caspase activity and evidence of mitochondrial dysfunction [42]. Quercetin, luteolin, kaempferol derivatives, and myricetin are flavonoids that have been shown to induce apoptosis and cell cycle arrest [43]. This is consistent with earlier reports that flavonoids and ellagitannins from pomegranate, such as quercetin, ellagic acid, and punicalagin, induce apoptosis and cell cycle arrest through mitochondrial signaling and PI3K/Akt inhibition [44]. Their results closely mirror our observations in *THP-1* cells and support the hypothesis that PPE-mediated apoptosis is driven by intrinsic mitochondrial signaling. Izuegbuna et al. further demonstrated dose-dependent cytotoxicity of native PPE against leukemia cell lines, with notable ROS generation, DNA fragmentation, and cell cycle arrest [45–48]. Although they did not investigate nano-formulations, their work confirms the baseline efficacy of crude PPE against hematologic malignancies. In our study, CDK8 downregulation provides additional insight into PPE’s cell cycle regulation, aligning with emerging evidence that CDK8 contributes to leukemic cell transcriptional elongation and oncogenic survival.

Interestingly, previous studies on ellagitannin-rich fractions from PPE showed cytotoxic activity on HL-60 leukemia cells with IC₅₀ values around 50 µg/mL and only moderate antimicrobial effects at high doses, suggesting that bioactivity is highly dependent on extract composition and formulation [49]. Our findings with PPE-EA and its nanoform demonstrate that specific solvent extraction combined with nano-encapsulation can dramatically improve biological efficacy across both cancerous and infectious targets [50].

In addition to its cytotoxic effects, n-PPE-EA modulated oxidative stress pathways in leukemia cells due to the presence of phenolic acids such as caffeic acid, ferulic acid, and sinapic acid, which may sensitize cancer cells to apoptosis via oxidative damage [51, 52]. It significantly increased intracellular catalase activity, a key antioxidant enzyme that neutralizes hydrogen peroxide, and reduced malondialdehyde (MDA) levels, a marker of lipid peroxidation and oxidative damage. This dual modulation of pro-oxidant and antioxidant balance is crucial in leukemia pathophysiology, where excessive ROS contribute to genomic instability but are also exploited by malignant cells to drive proliferation [53]. The observed antioxidant profile suggests that PPE-EA not only disrupts redox homeostasis in leukemic cells to induce cell death but may also provide protective effects to surrounding healthy cells by reducing systemic oxidative stress [25, 54].

Regarding antioxidant activity, our findings are supported by Bhandari et al., who observed increased catalase and reduced MDA in leukemic rats treated with PPE, indicating a restoration of redox balance [55]. Mo et al. similarly emphasized the superior antioxidant capacity of *Punica granatum* peel compared to other fruit-derived extracts, largely attributed to its rich polyphenolic and ellagitannin content [56]. These constituents, including punicalagin and ellagic acid, are known to regulate oxidative pathways and enhance enzymatic antioxidant responses, which were evident in our analysis of n-PPE-EA-treated cells [57].

The antimicrobial efficacy of PPE-EA and n-PPE-EA against *S. pyogenes* also reflects findings from previous literature. The observed antibacterial activity of PPE-EA may be attributed to polyphenol-induced membrane disruption and oxidative stress. Previous studies have reported that broad-spectrum antimicrobial activity of PPE, particularly against Gram-positive organisms, due to its phenolic constituents that disrupt bacterial membranes and inhibit protein synthesis [58, 59]. Spizzirri et al. advanced this understanding by showing that nano-formulated PPE produced larger inhibition zones and lower MICs compared to crude extracts [60], consistent with the enhanced antimicrobial activity observed in our study. In our experiments, nano-encapsulation resulted in significantly greater antimicrobial potency at lower concentrations, likely due to improved interaction with the bacterial surface and better retention of active compounds [16].

These results position n-PPE-EA as a promising candidate for integrative cancer therapy, especially in leukemia patients with high susceptibility to opportunistic infections. The dual action of n-PPE-EA addresses two major clinical challenges: tumor burden and infection risk within a single formulation [61]. This is particularly valuable in immunocompromised settings, where polypharmacy increases the likelihood of adverse drug interactions and patient non-adherence [16]. Moreover, the strong bioactivity at low concentrations suggests potential for targeted or topical delivery systems, such as mucosal gels or inhalable nano formulations, to prevent or treat infections during periods of chemotherapy-induced neutropenia [62]. The nano-formulation strategy applied here may also serve as a model for enhancing the therapeutic index of other phytochemicals with known bioactivity but poor pharmacokinetics.

The novelty of this work lies in demonstrating that nano-formulated pomegranate peel extract exerts synergistic cytotoxic and antimicrobial effects, supported by molecular docking and mechanistic validation. These findings extend beyond conventional phytochemical studies by linking bioavailability enhancement with clinically relevant dual activity, thereby offering a promising avenue for integrative cancer care strategies.

Nonetheless, several limitations must be acknowledged. The study was conducted in vitro, and the results may not fully reflect in vivo pharmacokinetics or toxicity. Only one leukemia cell line (THP-1) and a single bacterial strain (*S. pyogenes*) at a short-time point (48 h) were tested, which restricts generalizability. Protein-level validation was not performed, and specific phytochemicals were not isolated, limiting mechanistic precision. Future studies should therefore include multiple leukemia subtypes, additional bacterial and fungal pathogens, and in vivo models to validate safety and efficacy. Furthermore, targeted identification of the most active phytoconstituents, combined with protein-level and proteomic analyses, will provide deeper mechanistic insights.

## Conclusion

In conclusion, this study provides critical evidence that nano-formulated pomegranate peel extract exerts synergistic cytotoxic, antimicrobial, and antioxidant effects, offering a promising multifunctional candidate for integrative cancer care. By situating our findings within the context of existing literature and identifying key avenues for further exploration, this work advances the potential translation of PPE into therapeutic strategies for leukemia patients facing the dual challenges of malignancy and infection.

## Supplementary Information

Supplementary Material 1.

## Data Availability

Data will be made available on request.

## Abbreviations

AML: Acute Myeloid Leukemia
Aq: Distilled Water
BCL2: B-cell lymphoma type 2 gene
BHI: Brain Heart Infusion
BuOH: Butanol
cDNA: Complementary DNA
DLS: Dynamic Light Scattering
DMEM: Dulbecco’s Modified Eagle Medium
DMSO: Dimethyl sulfoxide
DOX: Doxorubicin
EA: Ethyl Acetate
EE: Encapsulation Efficiency
ESI: Electrospray Ionization
Etoh: Ethanol
FBS: Fetal Bovine Serum
Hex: Hexane
HMDB: Human Metabolome Database
IC₅₀: Half-maximal inhibitory concentration
LC-MS QTOF: Liquid Chromatography-Mass Spectrometry Quadrupole Time-Of-Flight
MDA: Malondialdehyde
MDR: Multidrug-Resistant
N-PPE-EA: Nano-Formulated Ethyl Acetate Extract Of Punica Granatum Peel
PBS: Phosphate-Buffered Saline
PDI: Polydispersity Index
PE: Petroleum Ether
PI: Propidium iodide
PI3K: Phosphoinositide 3 kinases
PPE: Pomegranate Peel Extracts
PPE-EA: Ethyl acetate fraction of pomegranate peel extracts
qPCR: Quantitative Real-Time PCR
RMSD: Root-Mean-Square Deviation
*S. pyogenes*: *Streptococcus pyogenes*
SD: Standard Deviation
TBARS: Thiobarbituric Acid Reactive Substances
