# Supplementary material for: Nano-formulated pomegranate extracts with dual cytotoxic and antimicrobial activity: molecular docking and mechanistic insights into leukemia cell targeting
Source: BMC Complement Med Ther. 2026 Feb 26;26:81. doi: 10.1186/s12906-026-05291-9 (PMC12955060; doi:10.1186/s12906-026-05291-9)
Supplement: Supplementary file 1 — Supplementary Material 1. [file 12906_2026_5291_MOESM1_ESM.pdf]

Cp No.	Name	Formula	Mwt	Rt	Conc. Ug/g
**1**	Kaempferol-3-O-rutinoside	C27H30O15	594.50	2.48	883.749
**2**	Luteolin	C15H10O6	286.24	2.51	2574.644
**3**	Chlorogenic acid	C16H18O9	354.31	2.53	20232.807
**4**	Nonadecene	C19H38	266.50	2.65	2857.530
**5**	Punicalin	C34H22O22	782.50	2.79	311.826
**6**	Naringenin	C15H12O5	272.25	3.26	6968.481
**7**	Gallic acid	C7H6O5	170.12	3.76	6013.795
**8**	Granatin B	C41H28O27	952.60	5.89	244.986
**9**	Punigluconin	C34H26O23	802.60	8.45	155.682
**10**	Kaempferol-7-O-glucosie	C21H20O11	448.40	9.39	120.006
**11**	Ellagic acid	C14H6O8	302.19	10.06	727.114
**12**	Quercetin	C15H10O7	302.23	10.52	4390.180
**13**	Dodecane	C15H26	170.33	10.78	201.714
**14**	Campesterol	C28H48O	400.70	11.18	13606.152
**15**	Myricetin	C15H10O8	318.23	12.16	158.956
**16**	Nonadecane	C19H40	268.50	13.22	332.717
**17**	Cyanidin-3-glucoside	C21H21ClO11	484.80	16.08	52.027
**18**	2,6,11-trimethyl-docosane	C25H52	352.70	16.19	116.723
**19**	Triacontane	C30H62	422.80	16.24	94.904
**20**	Apigenin	C15H10O5	270.24	18.48	90.039
**21**	Caffeic acid	C9H8O4	180.16	18.57	100.801
**22**	Sinapic acid	C11H12O5	224.21	19.09	528.322
**23**	Cyanidin-3-rutinoside	C27H31ClO15	631	20.37	62.153
**24**	Hexatriacontane	C36H74	507	20.58	119.753
**25**	Erucic acid	C22H42O2	338.60	20.80	61.218
**26**	Pelargonidin	C15H11O5N	287.24	20.87	187.952
**27**	Pedunculagin	C34H24O22	784.50	21.75	101.545
**28**	Punicalagin	C48H28O30	1084.70	22.38	44.311
**29**	Ferulic acid	C10H10O4	194.18	23.50	317.509
**30**	Lupeol	C30H50O	426.70	24.40	245.626
**31**	Punicic acid	C18H30O2	278.40	26.17	117.739
**32**	Epicatechin gallate	C22H18O10	442.40	27.58	335.378
**33**	9-Eicosene	C20H40	280.50	28.02	84.325
**34**	Copaene	C15H24	204.35	28.48	85.932
**35**	Heneicosane	C21H44	296.60	29.07	312.005
**36**	Squalene	C30H50	410.70	29.45	34.981
**37**	Stigmasterol	C29H48O	412.70	32.46	70.204
**38**	Gallocatechin	C15H14O7	306.27	32.63	61.147
**39**	Octacosane	C28H58	394.80	32.71	181.858
**40**	Castalagin	C41H26O26	934.60	32.87	24.417
**41**	Syringetin hexoside	C23H24O13	508.40	33.61	39.260
**42**	Betulinic acid	C30H48O3	456.70	34.39	235.178
**43**	Pelargonidin-3-rutinoside	C27H31ClO14	615.50	35.26	103.543
**44**	Pelargonidin-3-glucoside	C21H21ClO10	468.80	35.63	45.718
**45**	Eicosane	C20H42	282.50	35.78	74.152
**46**	Delphinidin-3-glucoside	C21H21ClO12	500.80	36.55	51.058
**47**	Tetracosane	C24H50	338.70	37.31	132.092
**48**	Gallocatechin gallate	C22H18O11	458.40	37.34	369.346
**49**	Rutin	C27H30O16	610.50	38.28	83.912
